# Health Effects of Drought: a Systematic Review of the Evidence

**DOI:** 10.1371/currents.dis.7a2cee9e980f91ad7697b570bcc4b004

**Published:** 2013-06-05

**Authors:** Carla Stanke, Marko Kerac, Christel Prudhomme, Jolyon Medlock, Virginia Murray

**Affiliations:** Extreme Events and Health Protection Section, Health Protection Agency, London, UK; Leonard Cheshire Disability and Inclusive Development Centre, Department of Epidemiology and Public Health, University College London, UK; Extreme Events and Health Protection Section, Public Health England, UK; Centre for Ecology and Hydrology, Wallingford, UK; Emergency Response Department, Public Health England, Salisbury, UK; Extreme Events and Health Protection Section, Public Health England, London, UK

## Abstract

Introduction. Climate change projections indicate that droughts will become more intense in the 21 century in some areas of the world. The El Niño Southern Oscillation is associated with drought in some countries, and forecasts can provide advance warning of the increased risk of adverse climate conditions. The most recent available data from EMDAT estimates that over 50 million people globally were affected by drought in 2011. Documentation of the health effects of drought is difficult, given the complexity in assigning a beginning/end and because effects tend to accumulate over time. Most health impacts are indirect because of its link to other mediating circumstances like loss of livelihoods. Methods. The following databases were searched: MEDLINE; CINAHL; Embase; PsychINFO, Cochrane Collection. Key references from extracted papers were hand-searched, and advice from experts was sought for further sources of literature. Inclusion criteria for papers summarised in tables include: explicit link made between drought as exposure and human health outcomes; all study designs/methods; all countries/contexts; any year of publication. Exclusion criteria include: drought meaning shortage unrelated to climate; papers not published in English; studies on dry/arid climates unless drought was noted as an abnormal climatological event. No formal quality evaluation was used on papers meeting inclusion criteria. Results. 87 papers meeting the inclusion criteria are summarised in tables. Additionally, 59 papers not strictly meeting the inclusion criteria are used as supporting text in relevant parts of the results section. Main categories of findings include: nutrition-related effects (including general malnutrition and mortality, micronutrient malnutrition, and anti-nutrient consumption); water-related disease (including E coli, cholera and algal bloom); airborne and dust-related disease (including silo gas exposure and coccidioidomycosis); vector borne disease (including malaria, dengue and West Nile Virus); mental health effects (including distress and other emotional consequences); and other health effects (including wildfire, effects of migration, and damage to infrastructure). Conclusions. The probability of drought-related health impacts varies widely and largely depends upon drought severity, baseline population vulnerability, existing health and sanitation infrastructure, and available resources with which to mitigate impacts as they occur. The socio-economic environment in which drought occurs influences the resilience of the affected population. Forecasting can be used to provide advance warning of the increased risk of adverse climate conditions and can support the disaster risk reduction process. Despite the complexities involved in documentation, research should continue and results should be shared widely in an effort to strengthen drought preparedness and response activities.

## INTRODUCTION

Reflecting awareness of the realities of climate change and the need to manage its wide-ranging effects^1^, there is currently great international interest by emergency coordinators and humanitarian assistance agencies in the impacts of extreme weather events and natural disasters on human health. While the health impacts of events such as flooding 2, heat waves 3 and wildfires 4 ^,^ 5 are relatively well described, there is limited evidence on the health impacts of drought.

While there are uncertainties about global-scale trends in droughts, the Intergovernmental Panel on Climate Change (IPCC) has identified some worrying projections on the future of drought events worldwide. Some regions of the world have experienced longer and more intense droughts (southern Europe and West Africa) while in other regions droughts have become less frequent, less intense or shorter (central North America and northwestern Australia)^1^, and it is more likely than not that human influence has contributed to the increase in droughts in the second half of the 20^th^ century 6. Because of reduced precipitation and/or increased evapotranspiration, there is medium confidence that droughts will become more intense in the 21st century in some areas including southern Europe and the Mediterranean, central Europe, central North America, Central America and Mexico, northeast Brazil and southern Africa. Elsewhere in the world there is low confidence because of inconsistencies in drought change projections 1. Table 1 summarizes both the observed changes in global-scale drought trends since 1950 and the changes projected in drought for the end of the 21^st^century.

**Table 1: Observed changes in drought trends regionally:** How confidence is defined Increasing levels of evidence combined with increasing degrees of agreement about the evidence are correlated with increasing levels of confidence. Low confidence: Low-medium available evidence, low agreement about the evidence Medium confidence: Medium-robust available evidence, medium agreement about the evidence High confidence: Medium-robust available evidence, high agreement about the evidence

Region	Observed changes in global-scale trends in droughts since 1950 1	Projected changes in drought for the end of the 21^st^ century 1
**North America**	Medium confidence that there has been an overall slight tendency toward less dryness, although analyses for some sub regions also indicate tendencies toward increasing dryness. Recent regional trends toward more severe drought conditions were identified over southern and western Canada, Alaska and Mexico, with subregional exceptions.	Low to medium confidence depending on the region. Medium confidence regarding increase in consecutive dry days and soil moisture anomaly in Texas and New Mexico; low confidence in other regions because of inconsistent change.
**Europe**	Medium confidence regarding increases in drynessbased on some indices in the southern part of the continent, but largeinconsistencies between indices in this region, and inconsistent or statistically insignificant trends in the rest of the continent.	Medium confidence: European area affected by stronger dryness (reduced soil moisture anomaly and consecutive dry days) with largest and most consistent changes in Mediterranean Europe.
**South America**	Low confidence because of spatially varying trends and inconsistencies between studies. For the Amazon, repeated intense droughts have been occurring in the last decades but no particular trend has been reported.	Low to medium confidence, depending on the region: inconsistent signal except for dryness increase (consecutive dry days and soil moisture anomaly) in north-eastern Brazil.
**Asia**	Low to medium confidence, depending on region. Low confidence in most regions due to spatially varying trends. Some areas have consistent increases but others display decreases in dryness indicated by different measures (soil moisture anomaly, Palmer Drought Severity Index, consecutive dry days). In East Asia, there is medium confidence in an overall tendency for increased dryness.	Low confidence because of inconsistent change in consecutive dry days and soil moisture anomaly between models in large part of domain.
**Africa**	Medium confidence in an overall increase in dryness, based on soil moisture anomaly and Palmer Drought Severity Index. For the Sahel, recent years have been characterized by greater interannual variability than the previous 40 years.	Low to medium confidence, depending on region. Low confidence in most regions, medium confidence of increase in dryness (consecutive dry days and soil moisture anomaly) in southern Africa except eastern part.
**Australia/ New Zealand**	Medium confidence: some regions with dryness decreases, others with dryness increases.	Low to medium confidence depending on region. Models agree on increase in consecutive dry days in South Australia, but inconsistent signal over most of South Australia in soil moisture anomaly. Inconsistent signal in consecutive dry days and soil moisture anomaly in North Australia. Strongest consecutive dry day increases in western half of Australia. Inconsistent change in area of drought depending on index used.
**Summary**	**There is medium confidence that since the 1950s, some regions of the world have experienced more intense and longer droughts (e.g., southern Europe, west Africa) but also opposite trends exist in other regions (e.g., central North America, north western Australia). There is not enough evidence at present to suggest high confidence in observed trends in dryness due to lack of direct observations and some geographical inconsistencies in the trends.** ****	**There is ** **medium confidence ** **in a projected increase in duration and intensity of droughts in some regions of the world, including southern Europe and the Mediterranean region, central Europe, central North America, Central America and Mexico, northeast Brazil, and southern Africa. Elsewhere there is overall ** **low confidence ** **because of insufficient agreement of projections of drought changes.**

The El Niño Southern Oscillation (ENSO) describes natural variations in the global climate system and involves changes in sea temperature and atmospheric pressure across the Pacific basin. ‘Southern Oscillation’ refers to above-average atmospheric pressures in the Indian Ocean associated with below-average pressures in the Pacific (and vice versa); ‘El Niño’ episodes refer to a phase of abnormally warm Pacific Ocean surface temperatures, while ‘La Niña’ episodes refer to a phase of abnormally cool ocean temperatures in the region. Both El Niño and La Niña phases are associated with spatial patterns of droughts and floods; pacific islands are strongly affected by ENSO variations, and an El Niño episode is usually accompanied by drought in southeast Asia, India, Australia, southeast Africa, Amazonia and northeast Brazil 1. Globally, disasters triggered by droughts are twice as frequent in the year following the onset of El Niño (warm events) than during other years 7. In areas where droughts can be anticipated, i.e., when linked with the El Niño Southern Oscillation, forecasts can be used to provide advance warning of the increased risk of adverse climate conditions and can support the disaster risk reduction process 8 ^,^ 1.

Documentation of the impacts of drought is difficult for a variety of reasons. Numerous indicators are used to capture and quantify the onset, duration, extent and beginning/ end of droughts 9. Droughts are slow-onset phenomena, which generally develop over an extended period of time and lack highly visible and structural impacts. They can be geographically extensive, affecting large areas regardless of geopolitical/country boundaries, and can exhibit complex spatial patterns. The societal impacts of drought can be slow to develop as they accumulate over time as the event continues, and the impacts can last for years. Finally, there is a lack of standardization in drought hazard characterization and in definitions of drought 10 ^,^ 11. The complexities of drought are reflected in over 150 published definitions 9 including:

- A prolonged dry period in the natural climate cycle 12;
- A period of abnormally dry weather long enough to cause a serious hydrological imbalance 1;
- An extended period of time characterized by a deficiency in a region's water supply that is the result of constantly below average precipitation 11;
- A temporary decrease of the average water availability due to rainfall deficiency 13.

Central to most definitions is a deficit of water from a ‘norm’ for a given spatial area. Drought can be further categorized based on how it is measured 9 ^,^ 14:

- meteorological drought, which is defined based on the degree of dryness and the duration of the dry period due to less precipitation than normal;
- hydrological drought, which is based on the impacts of precipitation shortages on surface or sub-surface (groundwater) water supplies;
- agricultural drought, which links characteristics of meteorological or hydrological drought to agricultural impacts, where the amount of moisture in the soil no longer meets the needs of a particular crop;
- socioeconomic drought, which occurs when the demand for a particular economic good exceeds supply as a result of weather-related shortfall in water supply and when water shortages begin to affect people.

Water source of interest is also key: meteorological drought is critical for rain-dependant areas such as the Sahel; agricultural (or soil moisture) drought is important for plant development; hydrological drought impacts on freshwater ecosystems, and groundwater drought for pumped water supply. While these different types of drought are not independent, they focus on physical processes of different complexity, scale and speed 15.

The impacts of drought are usually indirect. Being a slow-onset, long duration, spatially diffuse emergency, rather than a sudden, high-impact event (such as a flash flood or earthquake), drought differs from other natural hazards 16 and has many multiple ‘downstream’ effects. In the published literature, original links to drought are easily overlooked, e.g. wildfires are more common during times of drought, but resulting burn injuries are often attributed to the fire alone.

The effects of drought are critically dependent on context and underlying population vulnerability. Drought development and severity depend on the background level of water use (which might aggravate drought onset, duration and end) and infrastructure (which aims to mitigate the consequences of water deficit). The impact on health is particularly dependent on the socio-economic environment that can influence the resilience of the population. Poor health, poverty, and conflict are additional contributing factors to the impact of drought 17.

The most recent available data (covering drought disasters from 1900-2012) from EM-DAT, a worldwide disaster database maintained by the Centre for Research on the Epidemiology of Disasters, gives an indication of the devastating effects of drought on countries around the world. In 2011, it is estimated that 35 million people were affected by drought in China, while 17 million people were affected in Ethiopia, Kenya, Somalia, Uganda, Djibouti, Burundi and Niger. Droughts in the United States and Mexico were estimated to have resulted in $8 billion in damages, while droughts in China resulted in $2.4 billion in damages. Reported damages are often underestimated because of a lack of standardization methods for reporting and quantifying losses 11. For the period of 1900-2012, the countries with the greatest number of people affected by drought were India and China; for the same period, the countries with the greatest number of recorded drought-related mortalities were China, Bangladesh, India, Soviet Union, Ethiopia and Sudan 18.

Despite the challenges of describing the health impacts of drought, it is important to attempt to do so in order to better plan preparedness, mitigation, adaptation and response strategies. Systems already exist to predict drought 9 ^,^ 19; for these to benefit individual and societal wellbeing, it is important to know what impacts droughts might have. Actions trying to mitigate the worst effects can therefore be taken both in advance of and during a drought. Other reports have outlined some of the health impacts associated with drought in different contexts 20 ^,^ 21; this report offers the first systematic review of the global evidence on the known impacts of drought, aims to inform short-term drought-related policy and planning, and provides a basis for continuing research efforts.

## AIM AND OBJECTIVES

***Aim***

This review aims to support drought policy, planning and implementation of intervention strategies and adaptation measures by summarising available evidence of the health impacts of drought.

***Objectives***

- To systematically review published literature on the global health effects of drought;
- To explore contextual factors and population characteristics which contribute to different health outcomes;
- To identify gaps in knowledge to support further research efforts.

## METHODS

We conducted a systematic review of published literature following PRISMA guidelines 22. Search terms were adapted from previous publications on the health impact of related extreme events 2 (Box 1), and were also based on preliminary informal scoping studies of the literature.

**Box 1 - Search Terms Use:** 

**Exposure terms**
Drought* OR arid* OR rain*
Combined by AND with:
**Outcome terms**
Morbidity OR mortality OR health OR disease OR mental health OR injur* OR infect* OR diar* OR malnutrition OR water borne OR vector OR trachoma OR nutrition OR cyanide OR malaria OR schistosomiasis OR famine OR resp* OR typhoid fever OR amoebiasis OR cholera OR hepatitis A OR salmonellosis OR shigellosis OR dengue OR onchocerciasis OR Japanese encephalitis OR scabies OR impetigo OR conjunctivitis OR scrub typhus OR leptospirosis OR PTSD OR depress*

***Databases used***

The following databases were searched: MEDLINE; CINAHL; Embase; PsychINFO, Cochrane Collection. Key references from extracted papers were also hand-searched.

***Inclusion and exclusion criteria***

For extraction into summary tables, inclusion criteria were:

- Papers where an explicit link is made between drought as an exposure and human health outcomes, and where a health outcome is measured;
- All study designs and/or methods;
- All countries/contexts;
- Papers published in any year, until May 2012, based on individual database capabilities.

Papers which examined the link between drought and its impact on human health without explicitly quantifying or qualifying the impact are included in supporting text but not in the tables.

Exclusion criteria were:

- Drought as meaning shortage/deficiency unrelated to climate—as in ‘heroin drought’;
- Papers not published in English;
- Papers which discuss ‘dry season’ or arid conditions (unless drought was explicitly noted as an extreme event/unusual occurrence in the normal climate variability of the area).

Data from grey literature was not systematically searched for extraction into review tables, but sources including WHO, CDC, and advice from key experts are discussed in the accompanying text. Similarly, papers not fulfilling inclusion criteria were sometimes used to give a better contextual outline and are discussed in the relevant section.

**Study selection**

Full papers were identified following screening of all titles and abstracts by three study authors (CS, MK, and VM). These were further reviewed for eligibility and inclusion in the review by a single author.

**Data items, collection, synthesis and summary measures**

Data from relevant papers was extracted into summary tables. Column headings are: drought exposure; study design/sample; main health outcome measured; main results. Given the varied nature of studies and outcomes, meaningful quantitative summary statistics were not possible.

**Risk of bias and quality assessment**

Because drought is a natural phenomenon which cannot be induced experimentally, intervention studies were neither expected nor found. Most data was observational, and due to lack of data, we did not make any exclusions on the basis of study quality. Formal assessment of bias was not possible for each individual study.

**Analysis framework**

We classified the health impacts of drought thematically, based on the results of our search.

## RESULTS

Our search strategy identified over 6000 potential articles, of which 87 fulfilled inclusion criteria. Figure 1 summarises the study screening and selection process.

**Flow chart of literature search strategy:**
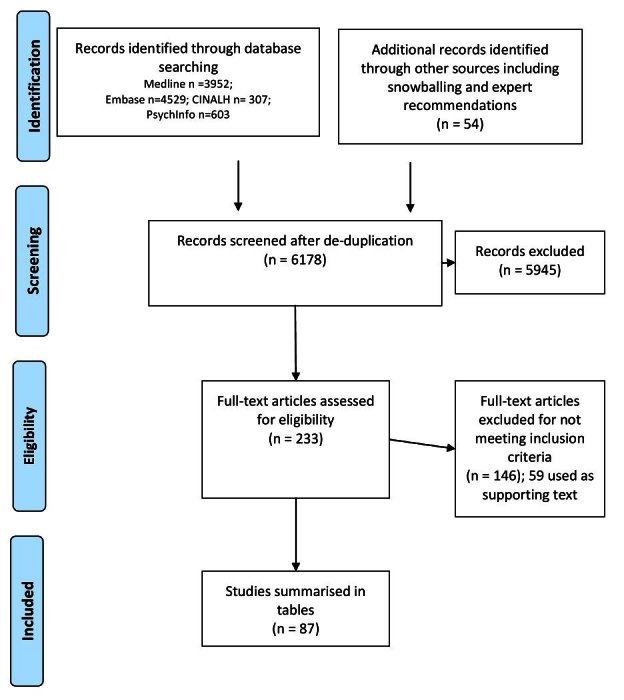

87 papers met our inclusion criteria and are summarized in Appendix 1: Tables 1-6. Additionally, 59 papers which did not meet our inclusion criteria yet offer supporting insights are included as text in relevant parts of the results section. Please see Appendix 2 for a full list of these supporting papers. Six categories of health effects were identified: nutrition-related effects; water-related disease; airborne and dust related disease; vector borne disease; mental health effects; and other health effects. Figure 2 represents the findings of our review and the categorizations used to classify the identified health effects associated with drought. Identified health effects follow a directional pathway: drought conditions affect the circumstances of living (i.e., water shortages and impacts on livelihoods); these circumstances can lead to a series of health effects, which results in increased morbidity and mortality. The factors linking drought with effects on health occur within the context of existing infrastructure (i.e., health, sanitation and other resources) and baseline public health (i.e., the capacity of populations to be resilient in the face of adverse conditions).

**Identified health impacts of drought and related influencing factors:**
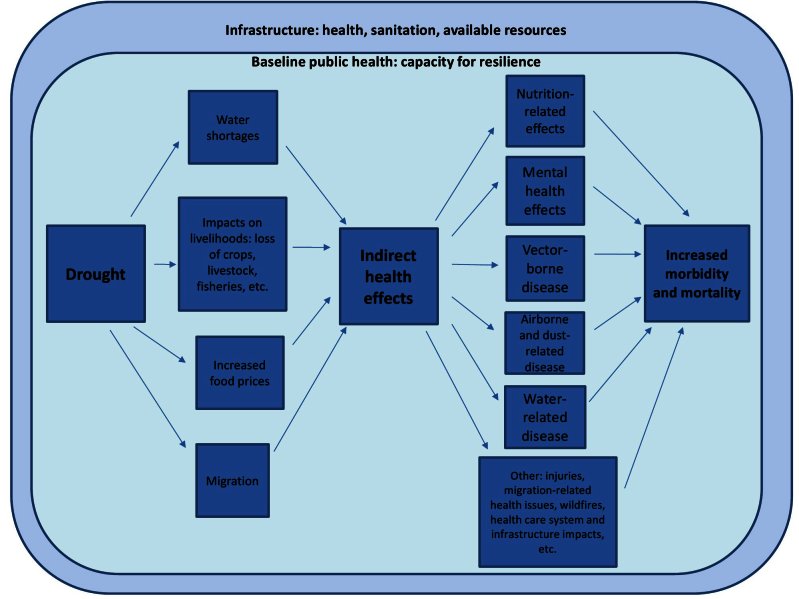

Figure 3 provides a visual geographic representation of papers identified in the review. As the health effects associated with drought are under-reported and under-documented, the papers (and contexts) included in this review should not be regarded as representative of the global drought and health problem, and do not provide a comprehensive representation of global drought events. Simple identification of the countries where studies were undertaken, rather than providing further detail on specific regions/cities, was chosen as the preferred method for delivering this information; more detail on each of the studies is included in Table 2.

**Geographic distribution of studies included in the review:**
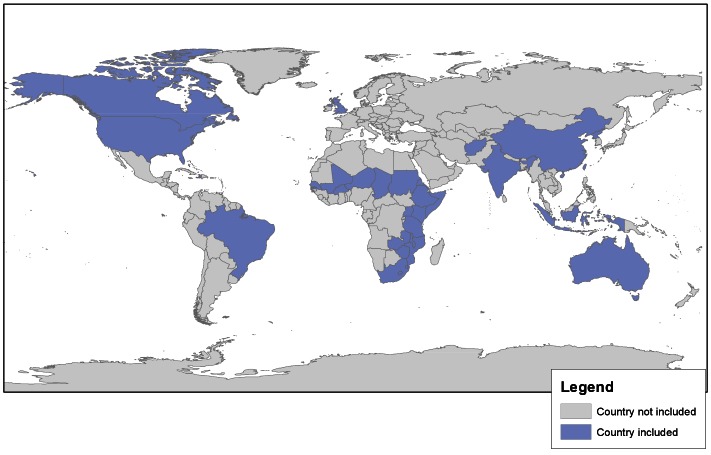

**1) EFFECTS ON NUTRITION**

***General malnutrition and mortality***

An impact on nutrition with implications for morbidity and mortality is the most obvious and best recognized health impact of drought 23 ^,^ 24 ^,^ 25. This is reflected by papers on these outcomes being the most numerous identified by our search: 49 papers are summarized in Table 2. The studies are all observational but vary in quality. Most are simple descriptions of population-level outcomes in the context of drought, some with a pre-drought baseline attempting to quantify magnitude of drought-related impact 26 ^,^ 27. Few attempt to measure individual exposures or account for obvious confounders such as household socioeconomic status or normal seasonal variations in nutritional status. Fewer still define the exact nature or details of the underlying drought. Settings, methods and outcomes are too varied to make quantitative synthesis meaningful, but strong qualitative messages do emerge.

Context and underlying vulnerabilities are critical. It is not by chance that the reports in Table 2 are from low and low-middle income countries. This does not mean that only they experience drought. Drought-related food shortage is only one aspect of the much more important concept of food security (defined as “…physical and economic access to sufficient, safe and nutritious food to meet dietary needs…” 28). Populations in resource rich developed countries do not usually experience drought-associated malnutrition because their populations have diverse diets sourced from geographically scattered suppliers: a production shortfall in one geographical area is easily made up for by another. Even in resource poor settings, “when famine is attributed to a natural factor, it is more likely true that the natural event is simply the triggering event among many contributing factors, rather than the main cause of famine” 24. Studies that do control for confounding also illustrate this point even in low-income settings. For example, crude analysis of 2004 data from Ethiopia shows greater mortality in drought affected areas 29. However, on multivariable analysis, drought drops out as a risk factor (p=0.8). In this study, the factors which did influence survival included household demographics, socioeconomic status, neighborhood characteristics, and receipt of food aid.****

The malnutrition/mortality impacts of drought are often indirect and complex. In the simplest case, drought affects ecosystems, thereby reducing food supplies (principally crops and livestock). This in turn reduces quantity and/or quality of nutrient intake, which leads to greater vulnerability to illness, which can increase mortality risk. Many studies in Table 2 show a high prevalence of malnutrition and/or mortality at the time of drought. This alone does not prove causality. Better evidence was from studies where spikes of malnutrition and mortality associated with drought were observed 30 ^,^ 31. Causes of mortality reflected prevalent conditions such as diarrhea, pneumonia and other infectious disease 32 ^,^ 33. However, while malnutrition and excess morbidity/mortality are directly and causally related 34, the relationship to the ‘upstream’ drought is neither straightforward nor linear and is influenced by multiple other factors. Illustrating the complexity:

- During a 1973-4 Ethiopian drought, distribution of malnutrition did not always reflect drought’s effects on food supply: the drought-affected North Ogaden area showed high malnutrition prevalence but the Issa desert did not. Issa, however, while having lowest rates of malnutrition among the areas studied also had the highest mortality rate 35.
- In another Ethiopian drought, type of economy was associated with manifestation of malnutrition: kwashiorkor (oedematous malnutrition) was not seen in agro-pastoralist areas 36.
- In a multi-country study in Southern Africa, areas which deteriorated most rapidly during drought included those which were better off pre-drought and those with high HIV prevalence 37.
- Drought’s interaction with HIV was different in different countries 38
- Not all population subgroups are equally vulnerable (see section below)

The potential effects of drought can be minimized by interventions. For example, several studies reported food aid being distributed at the time of nutrition surveys 39 ^,^ 40 ^,^ 41; some looked for and observed a protective effect 27 ^,^ 29 ^,^ 40. Less obvious but perhaps even more important are interventions at household level. One study from Kenya observed that toddlers (but not schoolchildren) were ‘protected’ from drought, seemingly by changes in family behavior and food distribution: carers lost weight but toddlers’ food intake did not change and they continued to grow 42.

***Micronutrient malnutrition***

Table 2b shows a smaller number of studies focusing on micronutrient deficit resulting from drought as the primary drought-related outcome. Some of the studies in Table 2a also note micronutrient deficit as a secondary outcome^41^^,^ 43 ^,^ 44 ^,^ 45 . Type of deficit varies and ranges from lack of iron causing anaemia to lack of vitamins (vitamin A) causing specific problems such as night blindness 41 ^,^ 46 and scurvy (vitamin C deficiency) 45 ^,^ 47. Subclinical deficit, detected by low blood levels of a particular micronutrient was, unsurprisingly, more prevalent than clinical deficit 47. Again, mechanisms are indirect and vary according to micronutrient: one study which attempted to correlate micronutrient deficit against drought severity found a link for some but not all micronutrients 41.

The message from these studies is that it is not only energy and protein content of diet that is important, but also quality and micronutrient content--the different types of malnutrition often co-exist 47. Specific risks in specific settings should be taken into account when planning drought-related interventions.

***Anti-nutrient consumption***

In resource poor settings, when a staple food crop fails and becomes less available following drought, people can be forced to make major changes to their usual diet 48. This can include eating less familiar foods or cutting corners in processing foods: Table 2c summarises some of the health risks can ensue. In Afghanistan in 1970-2, 22% of a drought-affected population developed veno-occlusive liver disease associated with eating wheat that had not been properly winnowed from a plant contaminant containing pyrrolizidine alkaloids 49. In India, drought was associated with aflatoxicosis due to mould-affected food, attributed to unseasonal rains prior to harvest and chronic drought conditions 50. In China, a drought caused selenium to enter the food chain causing problems which included hair and nail loss, skin lesions, tooth decay, and nervous system problems 51. In Ethiopia, consumption of drought-resistant grass pea led to irreversible neurodegenerative disease due to neurolathyrism 52. In Tanzania and Mozambique, outbreaks of Konzo, another serious neurological disease which causes irreversible paralysis, were due to inadequately processed cassava 53 54 ^,^ 55 ^,^ 56.****

***Malnutrition, vulnerable groups and drought***

Studies in Table 2 also demonstrate that not all subgroups within a population are equally vulnerable to the adverse effects of drought. This is likely true for all health outcomes, but is most clearly seen in the malnutrition/mortality papers. Risk is never equally distributed, but it is important to distinguish between unavoidable and avoidable excess risks. These can and often do co-exist. Understanding their role in different settings may help with targeting interventions to reduce adverse effects of drought.

Unavoidable risks are relatively constant across different drought contexts and are due to physiological vulnerabilities. This explains consistently higher malnutrition/mortality rates among infants 45 ^,^ 57 and young children compared to older children 29 and adults 26. It also likely contributes to lost pregnancies in Mozambique 58 and, to a lesser extent, to malnutrition among the elderly in India 59 ^,^ 60.****

Avoidable risks are more socially or culturally mediated. Examples include: rural-urban differences 61 and tribal and ethnic variations 62. Avoidable risks are particularly important to understand since they vary across settings and hence the same risk factor will require a different response depending on where it occurs. For example, boys are more vulnerable in some settings 58, girls in others 57; and socioeconomic gradients are seen in some 59 but not all studies 63.

**2) WATER-RELATED DISEASE**

Decreased water availability is a defining feature of most droughts. Related to this, water quality can also be affected 64. The relationship between water quantity and quality is complex 65. An important point, not always fully appreciated, is that both are necessary for good health 66. Seasonal variations in water related health outcomes are well-recognised 67 but whether drought should be considered as a prolonged dry season or a separate phenomenon altogether is a matter for debate.

As water discharge and water levels associated with droughts are typically low, dilution capacity is reduced and secondary impacts on freshwater systems emerge such as poorer water quality (e.g., higher concentration in chemical, nutrients and solid particles, lower dissolved oxygen) 68. Water quality can further worsen when intense rainfall follows a long dry spell (typically from convective storms associated with high summer temperature) and chemicals accumulated on the ground or roads wash out to the rivers 69. When dry spells are associated with anticyclonic conditions and high temperatures, this can speed up the development of droughts and increase the risk of secondary impacts. In particular, high temperatures result in high evaporative losses, drying up soil and plants; this can trigger agricultural droughts and increase wildfire risk. With dryer soils, infiltration capacity can also reduce and flooding risk can increase.

Also relevant to resource-rich countries as well as resource-poor is that people may switch their water source during drought, often to a lower quality supply. For instance, recent droughts in the UK have tempted people to consider private water supplies which are not under such direct control of water company drought restrictions 70. A risk of this is potentially poorer quality control: several outbreaks of infectious disease associated with private water supplies in England and Wales have been described 71. Recycling water is another option which people may resort to during drought: this too has risks for human health 72. Table 3 summarises studies which were explicit about drought linked to water-related disease. There were several categories of effect:

**Water-related disease caused by faecal/urine pollution**

Paucity of water sources during drought may increase the risk of remaining sources being contaminated by faces or urine: for instance, if an increased number of animals are drawn to drink by a river or borehole 73. Infected humans can also contribute to transmission around a water source: the more users there are, the more are at-risk and the more likely an outbreak of infectious disease. Associated risks might be further exacerbated if the water source in question is running at low levels; pathogens are more concentrated than normal 74 and it is more likely that anybody consuming the water would ingest a minimum infective dose.

Diseases which are transmitted by water and hence potentially affected by drought include amoebiasis, hepatitis A, salmonellosis, schistosomiasis, shigellosis, typhoid and paratyphoid (enteric fever). Evidence is, however, scarce. Few papers directly addressed this topic and were limited to drought-associated E.coli O157 75; cholera 76 ^,^ 77, and leptospirosis 78. In all these studies, it is important to note that drought was not the only exposure underlying the outbreak-- a whole chain of risk factors was responsible. Reflecting this upstream role, drought-related potential disease risk does not always translate to actual disease. In Haiti in 1976-1977, area-level comparison of severely and moderately drought affected areas showed no differences in reported diarrhoeal disease 79. Factors which did show an effect were: unemployed head of household; household socioeconomic status; large family size; quantity of water used (<19 litres/person/day).

**Water-related disease caused by poor handwashing/ hygiene**

Though it did not formally explore disease mechanisms, a 1978 study from Wales supports the plausible hypothesis that poor hand-washing (due to decreased water supply during drought) can also contribute to drought-associated diarrhoeal disease. Areas with the longest cuts to mains supply had significantly greater diarrhoea prevalence than areas with shorter-lasting or no daily cuts. Mains water meant that source contamination is an unlikely explanation for these results 80.

**Skin, eye and louse-borne disease that occur when there is lack of water for personal hygiene**

Skin infections associated with lack of water for washing include scabies 79, and impetigo – though we found no clear drought-associated references for the latter. Eye infections including conjunctivitis 79 were also associated with drought-related lack of water for washing.

**Disease caused by chemical and pollutant concentration**

Reduced water from a fixed source such as reservoir or lake concentrates its dissolved contents, including chemicals, metals and other pollutants. No studies directly examining the health effects of these as related to drought were found but there is certainly potential for adverse impact 81 ^,^ 82.

***Water-related disease caused by algae***

Following a drought in north-east Brazil in 1996, 126 patients in a haemodialysis unit developed signs and symptoms of acute neurotoxicity and hepatoxicity after water contaminated by cyanobacteria was used for dialysis; 60 subsequently died 83.

In the United States, after two dogs died drinking lake water contaminated by microcystin toxins, authorities launched a series of testing of cyanobacteria levels in lakes in Nebraska. High levels of microcystins were discovered in one lake, and failure to adequately notify the public resulted in more than 50 telephone calls to public health authorities complaining of skin rashes, lesions, blisters, vomiting, headaches and diarrhoea after swimming in the lake. Reduced lake levels as the result of a recent drought may have contributed to the increased cyanobacteria levels 84.

While not explicitly linked to drought, another study from Kenya discovered that, during the dry season, reservoirs used as a source of drinking water for humans showed microcystin levels of more than twice the WHO tolerable level for daily intake 85. These results are particularly worrying for countries which lack systems to provide safe drinking water on a widespread scale, and more research is needed to understand the extent of the problem.

**3) AIRBORNE AND DUST-RELATED DISEASE**

As soils become increasingly dry during a drought, dust circulated in the air is more likely. The United States dust bowl drought of the 1930s is a particularly well known example of this: hundreds and perhaps thousands of people who lived in the Great Plains died from “dust pneumonia,” a respiratory condition brought on by inhalation of excessive amounts of dust and dirt 86. Dust can be harmful via two mechanisms: pathogen carriage and direct trauma from inhaled particulates. In South Korea, one examination of dust clouds blown in by winds from the Mongolian desert found weak evidence of an association with mortality: a 1.7% (95% CI -1.6 to 5.3) increase in all cause deaths; a 2.2% (95% CI -3.5 to 8.3) increase in deaths among those aged 65 years and older; a 4.1% (95% CI -3.8 to 12.6) increase in deaths from cardiovascular and respiratory causes 87. A 2012 review of health impacts of desert dust found 50 studies describing a range of health effects including respiratory, cardiovascular and cardiopulmonary disease 88.

Table 4 summarizes reports linking drought to airborne/dust-related disease. Few studies were found making this link. Possible reasons for this lack of evidence include: 1) because the bulk of morbidity is in permanently dry (arid) areas, and our search did not include permanently arid conditions; 2) because the drought-dust link is often only implicit, not well enough described to be flagged by our search terms; 3) because the difference between an arid area and one affected by long term drought is a matter of degree and hence subjective. It is biologically plausible to assume that the health effects of drought-related dusts will be similar to those of dust in general 88 – possibly less severe for conditions where duration of exposure is relevant (assuming a drought is a time-limited event). As with previous sections, many of the respiratory effects of drought were indirect.

In a 1987-1989 Canadian drought, dust from a dried lake was associated with significant self-reported respiratory problems including cough and wheeze. Lung function did not, however, seem to be affected. Anxiety may have been responsible for symptom self-reporting, and short term exposure to dust may have been insufficient to cause measurable lung damage 89.

Another example of drought-related problems due to inhalation (this time of gas rather than dust) comes from case reports from New York. These described silo-gas exposure associated with the fact that a dry growing season raises nitrate levels in corn plants. As plants in silos biodegrade, they release nitrogen dioxide (NO2). Farm workers maintaining the silos were exposed to unusually high levels of NO2 resulting in hospitalization some cases: though important to note that this could have been fatal had levels been higher or exposure longer 90.****

Coccidioidomycosis (Valley Fever) shows the complex relationship with drought and other environmental events. It is caused by a fungus, Coccidioides, which lives in the soil of dry, low rainfall areas and is acquired by breathing in spores from the air. It can, for instance, be distributed during dust storms 91. Outbreaks in California were associated with heavy rains following a drought, possibly exacerbated by building work which disturbed the soil and released more spores than might otherwise have been the case 92. Another study on coccidioidomycosis was one of the few in this review which correlated drought intensity with increased incidence of disease, with a significant association (p<0.01) 93.

**4) VECTOR-BORNE DISEASE**

Our search revealed a number of papers which examined the impact of drought on various vector-borne diseases; eight met the inclusion criteria and are summarised in Table 5, and several others are used as supporting evidence in this section. The relationship between vector-borne diseases and climatological conditions is an important area to consider when planning public health drought mitigation strategies, and more research on each disease is needed to strengthen the existing evidence which, in many cases, is not definitive and quite sparse.

The mosquito is one of the most important arthropod vectors involved in the transmission of various vector-borne pathogens, and increased precipitation can cause mosquito densities to increase through provision of additional aquatic habitat. In a study of US wetlands that never dry compared to wetlands that dry annually, it was found that mosquito densities increase dramatically following natural drought events 94, and this was explained in the main through loss of competitors and predators thus allowing a rapid increase in mosquito numbers following re-colonisation resulting from re-wetting post-drought.

***Dengue***

An outbreak of dengue in1994 in Brazil was linked to a shortage of public water supplies as the result of a prolonged drought; increased mosquito abundance correlated with widespread household storage of water and with interrupted dengue suppression activities 95.

Given the prospect of climate change forecasts projecting increased drying conditions and decreased rainfall in Australia, projections of *Aedes aegypti* mosquito distribution were studied under current climate conditions and climate change scenarios for 2030 and 2050. Results indicate that the proliferation of domestic water storage tanks, used as an adaptation strategy during drought conditions, may expand the range of *Ae. aegypti* and create a high potential for dengue transmission during warm summer months 96. This phenomenon is likely to impact all urban mosquitoes that have adapted to exploit container habitats.

***Malaria***

Evidence of malaria associated with drought is mixed, and research on the effects of El Niño Southern Oscillation (ENSO) should be consulted to understand the global trends of drought patterns and their relationship to health.

One study which examined the link between malaria and ENSO found that malaria mortality is strongly related to drought in the year before outbreaks in Venezuela 97. Another study which examined ENSO and malaria epidemics in South America found that droughts are associated with malaria epidemics in Colombia (in the first year of an El Niño episode or in the year following the onset), Guyana (in the first year of an El Niño episode or in the year following the onset) and Venezuela (epidemics follow drought by one year) 98.

It is important to consider that some anopheline species exploit container habitats (water storage, or rainwater collecting habitats), while others require permanent water. The former applies to *Anopheles arabiensis*, and the latter applies to *Anopheles funestus*; both principle malaria vectors in Africa. The aquatic habitat favoured by the local malaria vectors is therefore crucial in determining the impact of drought on malaria.

In the Sahel, one study looked at *Anopheles funestus* and changes over time in malaria prevalence and incidence. In Senegal, between 1967 and 1992, parasite prevalence in children fell by 84%, and incidence fell by 82%. In Niger, malaria prevalence fell from 69% in 1969 to 23% in 1994 in the Niger River area; in Zinder, prevalence dropped from 89% in 1922 to 32% in 1994, and near Lake Chad, prevalence dropped from 40% in 1967 to 7% in 1996. The authors report that the decreases in malaria prevalence and incidence are likely due to the disappearance of the *A. funestus* as a result of severe droughts in the region 99. Another study of *Plasmodium falciparum* transmission by *Anopheles arabiensis* and *Anopheles funestus* during a period of drought (2004-2005) in Zambia reported reduced mosquito activity and reduced numbers of malaria cases during the period of drought. Numbers of *An. arabiensis* rebounded strongly when the rains returned in 2005-2006, while no *An. funestus* were collected during this time 100. The former is able to exploit the multitude of transient aquatic habitats following rain events with numbers increasing rapidly in the absence of predators and competitors.

Finally, highlighting the indirect nature of many of the health effects associated with drought, during a period of drought (1997) in the Irian Jaya province of Indonesia, two factors are believed responsible for a sharp rise in malaria cases (554 deaths in three months) amongst highland populations not normally exposed to malaria: severe food and water shortages in the highlands contributed to population migration to lower elevations where intense malaria transmission is common, and drought conditions resulted in numerous pools of standing water (normally fast-flowing), permitting a rapid increase in vector populations. The findings show a temporal association with ENSO-related drought and increases in malaria and associated deaths 101.

***St Louis encephalitis virus***

Evidence suggests that antecedent drought followed by wet conditions facilitated the amplification of the St Louis encephalitis (SLE) virus among *Culex nigripalpus* mosquitoes and a portion of wild birds in Florida, USA, resulting in subsequent transmission to humans 102 ^,^ 103. In another study water table depth was compared with human cases of SLE in Florida and results showed that May drought occurred in 83% of the areas studied prior to the report of at least one human case of SLE 104. This study builds on the previous studies and provides further evidence that antecedent drought and near-coincident wetting are significantly associated with human SLE cases. *Culex* mosquitoes tend to exploit transient water bodies and can proliferate in the absence of predators.

***West Nile Virus***

A number of studies have identified links between human West Nile virus (WNV) and drought in the United States. In a study comparing transmission patterns of WNV in California from 2004-2007, increases in human infection in 2007 were associated with severe drought conditions as well as other surveillance indicators including neglected swimming pools 105. In a study of human WNV incidences compared with annual rainfall levels in Mississippi, an inverse relationship was identified between precipitation levels of the previous year and the relative risk of human WNV, suggesting that drought acts as a potential mechanism for increased risk of human WNV transmission; given climate change projections for increased drought in certain areas of the world, the authors believe that the risk of human WNV will also increase 106. In western Colorado, dry spring and summer conditions appear to increase the risk of human WNV infection 107. In the eastern US, another study found that WNV proliferates under drought conditions 108. Similar to findings about SLE, one study found that widespread drought conditions in the spring, followed by a wetter summer, increase the probability of human WNV cases 109.

***Rift Valley fever virus***

Transmission of Rift Valley fever virus (RVFV) is reported to be epizootic during wet years, especially following droughts; uncontrolled air travel could introduce RVFV to North America or Europe where susceptible hosts and suitable vectors reside, and could potentially have serious impacts on human health 110. The virus survives in the eggs of *Aedes* mosquitoes. These mosquitoes survive out of water in areas prone to flooding, such as dambos or depressions, which are their main aquatic habitats in West Africa. Following heavy rainfall events, these habitats flood, and the resulting infected adults (from infected eggs) emerge to transmit the virus to animals. The pathogen is then passed from animals onto the local *Culex* populations precipitating a much larger outbreak. Flooding following drought results in large-scale simultaneous hatching of infected eggs and thus simultaneous outbreaks of RVFV across Africa.

***Japanese encephalitis***

One study which examined an epidemic of Japanese encephalitis in Andhra Pradesh, India, noted that drought conditions existed before the epidemic started in the sample population; however, this was not explored thoroughly as a potential risk factor which contributed to the outbreak 111.

***Chikungunya***

One study which used satellite data and epidemiologic investigation to examine epidemics of chikungunya in Kenya found that unusually warm and dry conditions preceded outbreaks of chikungunya (63% of population tested), suggesting that drought-affected populations may be at a higher risk for chikungunya infection 112. The study’s small sample size and lack of other studies to compare make it difficult to generalize findings. Throughout its range chikungunya is transmitted by *Aedes* mosquitoes that exploit container habitats in urban areas, so water storage during drought is likely to favour these vectors and transmission of this virus.

***Tick-borne disease***

A study measuring both the prevalence and incidence of tick-borne borreliosis in Senegal reports an average annual incidence rate of 5.1% in the sample population and 0.09% prevalence in a different sample population. The authors attribute the persistence of sub-Saharan drought as a possible source responsible for the spread of the *Alectorobius sonrai* vector to new areas of West Africa 113 ^,^ 114; further investigation is necessary to confirm these findings.

Another study of tick-borne relapsing fever from the same site in Senegal, this one a 14 year longitudinal study, found an average incidence rate of 11 per 100 person-years; the authors also attributed the ongoing sub-Saharan drought as a factor responsible for the spread of the

*Ornithodoros sonrai* vector to the West African savannah 115. Again, more research is necessary to understand the possible relationship between drought and this disease. As the majority of tick species in more temperate parts of the world are reliant upon retaining their body moisture content, drought conditions would not tend to favour such ‘forest-species’. However, in arid areas, the effect of drought is likely to be much less.

***Schistosomiasis***

One study which compared the prevalence of *Schistosoma haematobium* in coastal Kenya in 2000 and 2009 recorded the disappearance of schistosomiasis infection clusters and a slight decline in prevalence from 55.7% to 43.2% following periods of prolonged drought 116. *Schistosoma* parasites require an aquatic snail as a vector, hence drought probably reduces the number of these snails and reduces the transmission potential. Furthermore, humans become infected when standing in water with infective metacercariae, therefore less available water during a drought will mean a lower exposure to the pathogens.

***Chagas disease***

One study examining an unusually high incidence of Chagas disease in Brazil reported that drought conditions may contribute to outbreaks in humans, yet no information was provided to give evidence of the link between the two 117.

**5) MENTAL HEALTH**

Research suggests that drought contributes to the business-related pressures that farmers must face, with severe drought resulting in financial impacts and consequent emotional stress 118. A number of studies, mainly from Australia, have documented the impacts of drought on mental health. Distress or ‘emotional consequences of drought’ (or similar terminology) were the indicators most often measured as drought-related mental health outcomes; depression, anxiety and post-traumatic stress disorder were not often measured. Most studies utilized focus group or one-to-one interviews as the main study design methodology, with small sample sizes; postal surveys and self-report questionnaires were also used. Of the 16 papers summarized in Table 6, three focused on the effects on children and the remainder focused on adults; most studied rural populations whose livelihoods (i.e., farming) are environment-dependent. A full list of mental health papers identified through our search is available in Table 6.

Not surprisingly, results indicate that prolonged drought does indeed have negative impacts on the mental health of the populations studied. For those studies which examined the effects of drought on children, worry about family 119 ^,^ 120 and feelings of loss 120 ^,^ 121 were common identified themes.****

Those studies that examined the mental health impacts of drought on farmers yield a number of findings that support the idea that drought negatively impacts on mental health. Drought-related stress was identified as ‘high’ in 71.8% of farm workers in one study 122; another study reported that 34% of participants experienced stress ‘fairly often’ or ‘very often’ 123, and other studies report physical symptoms associated with stress (including disturbed sleep, crying, being tired) 124 ^,^ 125 ^,^ 126. One study found twice the rate of mental health problems in farmers currently in drought compared to farmers not currently in drought 127.

Other broad findings from all the studies include increased alcohol consumption 122 ^,^ 126 ^,^ 128 and various experiences of loss or worry 119 ^,^ 120 ^,^ 124 ^,^ 126 ^,^ 129 ^,^ 130 ^,^ 131 as a consequence of drought. One study from Brazil compared anxiety levels of drought-affected communities with drought-free communities and found that study participants from drought communities had significantly higher levels of state and trait anxiety than drought-free participants. Participants from drought communities also scored significantly higher in emotional distress than drought-free participants 132.

A related study which examined the impacts on dryland salinity (defined as salinity in un-irrigated landscapes) on the mental health of populations living in the rural south-western corner of Western Australia found that living in areas of dryland salinity was associated with increased relative risk for hospitalization for depression. The authors liken these findings to those of drought in that salinity of farmland results in loss of farm productivity and decreased land values with subsequent increases in financial pressures 133.

The available evidence suggests that drought has negatively impacted the mental health of people in rural communities whose livelihoods often depend on rainfall.

***Suicide***

While higher rates of suicide have been observed in rural farming communities in Australia compared to non-farming communities 134, an explicit link to drought as a causal factor has not yet been established. In a study which examined rates of suicide in farmers during a prolonged drought (2001-2007) in Victoria, Australia, there was no evidence of a pattern of increased farming suicides during the drought years studied. However, drought as a risk factor that contributes to stress in rural communities should not be dismissed 135. Another study identified suicide or suicidal thoughts as a result of drought-related stress yet no quantification of this was reported 128.

While most of the studies offer good qualitative insights into the emotional consequences of drought, more rigorous study is needed to measure the potential impacts of drought on mental illnesses, including depression, anxiety and suicide, in order to determine the extent of the problem and to determine if findings are applicable to other contexts. It is also important to note that most of the mental health studies included in this review were conducted in a wealthy country context (i.e., Australia); more studies from different settings are needed to help understand the impacts of drought on mental health in other populations.

A number of interventions have been implemented to support the mental health of rural populations affected by prolonged drought in Australia, which suggests that local authorities recognize the magnitude of the problem and the importance of addressing it 136 ^,^ 137 ^,^ 138 ^,^ 139 ^,^ 140.

**6) OTHER HEALTH EFFECTS**

A number of other health outcomes have been associated with drought. See Table 7 for more detailed information on studies that met the inclusion criteria of the review.

***Injury***

Drought can cause rivers, lakes and streams to have lower than normal levels of water. Diving into these bodies of water can result in injury, which was observed following a drought in Wisconsin, USA, in 1988. Seven people sustained spinal cord injuries after diving in familiar sites they believed were deeper than they actually were 141 ^,^ 142. More research is necessary to understand the extent of this problem. During high heat and drought conditions it is important to monitor water depth levels in popular recreation areas and provide adequate public health messaging about the potential health hazards associated with diving.

**Cancer**

Using GIS mapping technology to compare esophageal cancer mortality and drought indices in China, studies suggest an association may exist between areas with high drought index and high esophageal cancer mortality. Possible reasons given include high salinity of water in water-scarce regions, which promotes nitrosamine (a carcinogen of esophageal cancer) formation in plants grown in the area, and fumonisin B1, another toxin which can contaminate corn; both of these toxins can enter the body through the food chain. Lifestyle and genetic factors should not be ignored 143 ^,^ 144.

***Nomads***

The effects of drought on the health of nomads have not been well-documented. Nomadic populations live in close proximity to livestock and as such are susceptible to parasites transmitted by animals. Drought conditions may cause nomadic people to abandon their lifestyles and settle in urban areas, which can be accompanied by a host of potential disease threats including sexually transmitted infection transmission, crowded living conditions and poor sanitation 145. More research is needed to understand the relationship between drought and health on populations who regularly migrate to sustain their livelihoods.

***Zoonotic disease***

Our search revealed one study which identified El-Niño-related drought as one of the factors which contributed to the spillover of Nipah virus from pig populations to human populations in Malaysia, yet details of how drought conditions may have affected the outbreak are not clear 146. More research is needed in order to understand how drought may influence zoonotic pathogen outbreaks in humans.

***Heat wave and Wildfire***

In temperate areas, long droughts can be associated with heat waves during the summer, especially when anti-cyclonic conditions (high pressure usually associated with clear skies and in the summer increased temperature due to increased radiation) remain on land masses over an extended period. This was the case in August 2003 when Western Europe experienced some extreme temperatures 147: 70,000 additional deaths occurred during the summer period, with over 30,000 of these occurring during the heatwave, and over 25,000 wildfires were recorded 148 ^,^ 149.

The lack of moisture in the soil during droughts could also amplify surface temperature anomalies 150. With reduced soil moisture and increased temperatures, plants will lose some of their water content; they might transpire less to mitigate the lack of water but this is an aggravating factor for wildfires.In 1996, the Panhandle region of Texas (United States) experienced severe wildfires which resulted in the deaths of 12 people. Circumstances were ideal for the wildfires as the area had experienced “extreme” drought conditions in the five months prior to the fires 151. Above-average temperatures in Russia in 2010 were accompanied by extensive wildfire outbreaks in 22 regions across the Federation 152; in an analysis of the effects of heat on mortality, 54,000 cumulative excess deaths in July and August of 2010 were observed compared to the same period in 2009 153.****One review has documented the human health effects of wildfires with links to high heat (and likely drought conditions) 154, and further investigation and documentation are warranted.

***Migration***

In certain regions of the world, drought contributes to the mass migration of people fleeing conditions where sustaining livelihoods is no longer possible. The literature on the impacts of drought-related migration is vast, and given the complexities potentially involved (climatological conditions, conflict, baseline health of populations, sanitation and living conditions in refuge areas, community resistance to infections, etc.), it is difficult to identify drought as the sole exposure variable linked to a given health outcome.

One paper examined the health effects of the 1984-85 resettlement program in Ethiopia where 600,000 drought victims migrated from north and central Ethiopia to western Ethiopia 155. People who re-settled came from the malaria-free highlands to the humid western region of the country where local people have more resistance to malaria infection, and increased malaria mortality rates were reported in the settlements. Higher rates of intestinal parasites (associated with high population density and poor sanitation) were seen in the settlers, and people were exposed to onchocerciasis for the first time upon re-settlement. Cases of trypanosomiasis, sand flea infestation and nonfilarial elephantiasis were reported in settlement areas, as were cases of severe malnutrition with high mortality rates. Mental stress was observed in both settlers and in indigenous populations where settlement populations were based; unsurprisingly, reasons included family separation, uncertainty, and hostilities towards settlers.

Following the most severe drought ever recorded in Brazil, thousands of people migrated to the city of Fortaleza in 1878. Smallpox was introduced to the city through a sailor, and the disease quickly spread through the drought re-settlement communities; over 35,000 people died of smallpox in the span of four months 156. Yet again, this study illustrates the indirect effects that drought may have on public health.

Reports of a drought-induced migration of pastoralists in West Africa in the 1970s note exacerbation of cholera in settlement communities given the conditions of the relief camps (poor sanitation, poor water supplies, poor nutrition) 157.

Observational accounts of a drought-induced migration in Sudan note the impoverished living conditions in migrant communities, including poor sanitation systems, unsafe drinking water and inadequate health services. Diarrhoeal disease, malnutrition, typhoid, hepatitis, meningococcal meningitis, and cutaneous leishmaniasis were some of the diseases observed in those who migrated 158. Certainly there are countless reports of drought-induced health problems related to migration similar to this one, and continued effort should be made to document the health issues indirectly related to drought.

***Damage to healthcare system***

Surprisingly, our search revealed virtually nothing on the impacts of drought on healthcare services and facilities. One report offering anecdotal observations from a rural hospital in east Swaziland following ‘the worst drought in living memory’ noted overcrowding in the hospital and increased workload of staff 159. A combination of the limitations of our search and lack of documentation of the ‘downstream’ effects of drought, i.e., impacts on healthcare services, may contribute to the lack of literature on this topic.

***Damage to infrastructure***

Drought can affect the production of electricity when water levels in lakes, rivers or reservoirs fall below intakes used for drawing water for cooling of power plants, and again when surface water becomes too warm to be used for cooling 160. The obvious indirect effects of disrupted power supplies involve those people who rely on electricity to sustain their health, i.e. the use of medical equipment or refrigeration of medications. From a report summarizing the 2003 heat wave in France, the impacts of the extreme heat on water levels in dams resulted in hydroelectric energy not able to be used as it should be because of reduced water levels, likely an effect of the drought-like conditions of reduced rainfall combined with excessive heat 161. A recent report concluded that thermoelectric power production in southern and southeastern Europe and the southeastern US will be affected by climate change through a combination of increased water temperatures and reduced river flows, especially during the summer 162.

The health effects of disrupted electricity as a result of drought, particularly on vulnerable groups dependent upon power to sustain their health care needs, deserves significant further research.

## DISCUSSION

There is no doubt that drought has multiple adverse indirect health outcomes; our review illustrates how varied and wide-ranging these are, and serves as a call for policy makers and funders to treat drought as seriously as other more dramatic and visible extreme weather events. To do this, they should consider they key message permeating almost all our studies: that underlying population and individual vulnerability/resilience factors are critical.

The probability of drought related health impact varies from high (e.g. risk of drought-associated malnutrition in a resource-poor developing country) to negligible (e.g. risk of drought-associated malnutrition for wealthy individuals in the same developing country, or for developed country populations), based on the drought characteristics, existing infrastructure, and available resources with which to mitigate health impacts as they appear.

The severity of impact also varies according to underlying vulnerability: from death at one extreme to minimal or no effect where the population/individuals have financial and other resources to buffer themselves from any potential effects.

As well as the categories of impact presented in our results section, it is useful to summarise our findings according to another framework outlining the long terms impacts of disasters on community health 163. This helps put drought in context with other natural hazards and identifies the need for enhanced disease surveillance following disasters in order to better capture those health impacts which manifest in the longer term. Five categories of long-term health effects associated with natural hazards were identified:

- Effects of malnutrition and trace element toxicity

These effects are exclusive to populations vulnerable to food insecurity and poverty in resource poor developing countries.

- Infectious disease risk

Vulnerable populations are most at risk, but our review shows some evidence that even in developed countries with high quality water sources, drought-related behaviours such as worsened hand washing can result in spread of infectious disease. Of particular concern is population displacement and the often-associated risk of increased infectious disease transmission.

- Chronic systemic illness

Chronic systemic illness is most associated with interruptions in the delivery of health care, which may be affected by disruptions in electricity supply.

- Chronic disability/pain following physical injury

Though not a major feature of drought, examples include the neurological diseases due to toxin ingestion and spinal cord injury due to diving into shallow water.

- Mental health outcomes

This is a universal feature of drought, mainly for people whose livelihoods depend on rainfall or who are forced to migrate. Even though developed country populations are not at risk of drought’s most severe consequences, the impact of drought on certain occupations/livelihoods and the stress that it causes is not to be underestimated. Mental health outcomes are not specific to drought but do underlie the point that psychological wellbeing is a critical part of health.

Other issues associated with drought and health include regulated vs. unregulated water supplies. In developed countries, public water supplies are tightly regulated and quality controlled; populations without widespread access to regulated drinking water supplies may be particularly vulnerable to water-related health effects. A related issue is that of public vs. private water supply. In the UK, for example, 1% of the population relies on private water supplies which are not managed in the same way as public water supplies, and water-related health effects have been documented 70; as people may seek alternate water supplies during times of drought, this may be an issue for both developed and developing countries. Population growth and urban expansion place additional stress on local water supplies in many places, potentially exacerbating competition for already-scarce water resources 164. It is vital not to confuse the current ‘absence of evidence’ regarding health and drought with ‘evidence of absence’ of health impacts—a relative lack of documentation on the health impacts of drought should not be interpreted to mean that drought has no effect on human health.

The Hyogo Framework for Action (HFA) is a major international initiative to build the resilience of nations and communities to disasters 165. HFA actions include:

- ensure disaster risk reduction is a national and local priority;
- identify, assess and monitor disaster risks and enhance early warning;
- use knowledge, innovation and education to build a culture of safety and resilience;
- reduce underlying risk factors;
- strengthen disaster preparedness for effective response at all levels.

To be effective, these actions require strong underpinning evidence to identify the known impacts associated with each hazard.

Drought preparedness measures are key and should incorporate the wide-ranging impacts that drought may have on human health. Drought policies, based in local contexts, should emphasize prevention, preparedness and mitigation rather than relying solely on crisis management as actions can be taken before a drought to minimize the potential effects on people. Essential to drought preparedness, and therefore of use in reducing the effects of drought on human health, are early warnings and drought monitoring systems (to warn people of potential threats to wellbeing, and to create a historical record to assess changing conditions), and risk identification of vulnerable population groups, regions, and sectors most at risk of the effects of drought 166. Additionally, further adaptation strategies exist to strengthen health sector preparedness for drought, including: heat-health action plans; emergency medical services; improved climate-sensitive disease surveillance and control; safe water and improved sanitation 167. In an example from Vietnam, health-related drought adaptation measures included provision of first aid training (for treatment of diarrhoea and respiratory diseases), raised awareness of nutrition, improved access to clean water, and provision of hygiene kits 166. In the case of drought-related effects on nutrition, preparedness can be strengthened with the knowledge that improvements in nutritional status may best be achieved based on an understanding of the local causes of malnutrition, as poor nutrition may be a chronic or structural problem independent of drought in a given area 168.

**Limitations**

Due to its slow onset and association with other emergencies, most health effects of drought are indirect and therefore under-investigated, under-recognised and under-reported. As it is difficult to identify drought as the sole exposure responsible for a given health outcome, it is likely that this search did not locate all published studies.

The studies included in the tables were all observational studies with widely varying study designs, of varying quality levels. However, since it is unethical to conduct experimental studies on this topic, good quality observational studies must continue to be done.

Some health outcomes, such as ‘injuries,’ yielded very few studies, and as such it is difficult to generalize the findings and extrapolate results to other settings; caution is needed in interpretation of these results, and more research is needed on several topics in order to strengthen the evidence base.

Reporting bias is very likely (as severe droughts are more likely to be reported and associated with adverse health outcomes), which is one of several reasons why quantitative meta-analysis is neither possible nor sensible in order to quantify impact of drought. Important lessons on how to successfully manage drought to avert/minimise health effects may have been missed.

This review did not address conflict, either as an exposure variable (i.e., in the case of countries engaged in conflict, with co-existent drought as an additional variable) nor as a health outcome (i.e., in the case of countries engaged in ‘water wars’ over scarce water resources, with conflict-related consequential impacts on human health). The issue of conflict is too complex to be included in a general overview of the literature as presented here, and the complicated relationship between drought and conflict deserves further attention.

Finally, this is a systematic but not exhaustive review, which included results from peer-reviewed articles only in the final analyses; there are many other valuable reports in grey literature (sources may include World Food Programme; Food and Agriculture Organization of the United Nations; the International Fund for Agricultural Development, and the Joint United Nations Programme on HIV/AIDS, among others), in other languages, or found under other search terms such as ENSO. However, these are extremely unlikely to change overall conclusions and interpretations, and may be lower quality than the peer review published reports which were found.

**Calls for the future**

Although this review represents an initial attempt at summarising the available evidence on the global health effects of drought, a number of suggestions for improved documentation were identified:

- Drought with clear identification as a keyword/risk factor
- More systematic reporting of drought impacts
- Standardization of definitions to facilitate future trend analysis and meta-analysis / comparative analysis.
- Good baseline data to facilitate better understanding of the impacts of drought, the role of drought plans and drought mitigation strategies

Recommendations for research into other areas of drought include:

- Links with other extreme events, especially flood, heat and wildfires
- Long term impacts of malnutrition (reflecting the Barker hypothesis of how nutrition influences heart health) (http://www.thebarkertheory.org/index.php);
- Alcohol use and its relationship to sufferers of drought;
- Gender differences in experiences of drought-related mental health problems;
- The impacts of disrupted electricity supply on the health of vulnerable populations;
- The impacts of drought on vector borne disease;
- The impacts that drought adaptation strategies may have on human health (i.e., storing rain water and implications for increased vector borne disease in some settings);
- The complex relationship between drought, conflict and consequent impacts on human health. Related to this may be issues of migration and its enormous influence on health.

## CONCLUSIONS

This review provides valuable evidence which will support emergency coordinators and international assistance agencies in efforts to plan for and respond to drought emergencies.

Health effects identified in the review include nutrition-related effects (including general malnutrition and mortality, micronutrient malnutrition, and anti-nutrient consumption); water-related disease (including E coli, cholera and algal bloom); airborne and dust-related disease (including silo gas exposure and coccidioidomycosis); vector borne disease (including malaria, dengue and West Nile virus); mental health effects (including distress and other emotional consequences); and other health effects (including wildfire, effects of migration, and damage to infrastructure). Individual and population vulnerability and resilience factors are critical in exacerbating or mitigating any drought-related impact. Understanding these provides valuable opportunities for enhancing resilience at both national and community levels, reflecting the call of the Hyogo Framework for Action.

Forecasting can be used to provide advance warning of the increased risk of adverse climate conditions and can support the disaster risk reduction process. In the short to medium term, robust warning and response systems available to resource-rich developed countries often mitigate many of the worst adverse health effects of drought. The downside of this success is that the health risks of drought may be insufficiently appreciated in these contexts. In the longer term, climate change is likely to lead to more frequent and severe droughts in certain areas of the world. Even wealthy countries’ drought management systems may eventually become overwhelmed and as this happens, the impacts of drought could become correspondingly more marked. Consideration of the existing evidence on the health risks associated with drought, combined with continued efforts to improve research methodologies to record these complex health effects, will support drought policy, mitigation and adaptation measures locally, nationally and internationally.

## Competing Interests

The authors have declared that no competing interests exist.

## Correspondence

Email: Carla.stanke@phe.gov.uk

## Appendix 1

### Tables 2-7

Download PDF

## Appendix 2

### Papers *not* in tables, used as supporting text in Results section

**Table:**

Scrimshaw, 1987^23^ Yip, 1997^24^ Taye et al., 2010^25^ Black et al., 2008^34^ Lockett and Grivetti, 2000^48^ Moran et al., 2004^64^ Gundry et al., 2004^65^ Askenazy et al., 2003^66^ Vargas et al., 2004^67^ Said et al., 2003^71^ O’Toole et al., 2007^72^ Shehane et al., 2005^73^ Kuntz and Murray, 2009^74^ Snyder and Benotti, 2010^81^ Soller et al., 2005^82^ Walker et al., 2008^84^ Mwaura et al., 2004^85^ Cook et al., 2007^86^ Kwon et al., 2002^87^ De Longueville et al., 2012^88^	Zender and Talamantes, 2006^91^ Chase and Knight, 2003^94^ Pontes et al., 2000^95^ Beebe et al., 2009^96^ Bouma and Dye, 1997^97^ Gagnon et al., 2002^98^ Kent et al., 2007^100^ Shaman et al., 2002^102^ Shaman et al., 2003^103^ Shaman et al., 2004^104^ Wang et al., 2010^106^ Shaman et al., 2010^107^ Deichmeister and Telang, 2011^108^ Shaman et al., 2005^109^ Weaver and Reisen, 2010^110^ Rao et al., 2000^111^ Barrett et al., 1979^117^ Fragar et al., 2008^118^ Speldewinde et al., 2009^133^ Sartore et al., 2005^136^	Sartore et al., 2007^137^ Sartore et al., 2008b^138^ Fuller et al., 2007^139^ Hart et al., 2011^140^ Page and Fragar, 2002^134^ Raymond, 1988^142^ Wu and Li, 2007^143^ Wu et al., 2008^144^ Macpherson, 1994^145^ Chua, 2010^146^ UNEP, 2004^148^ CDC, 2007b^151^ Finlay et al., 2012^154^ Prothero, 1994^157^ Ahmed, 1990^158^ Wright and Ford, 1992^159^ NETL, 2009^160^ Letard et al., 2004^161^ Van Vliet et al., 2012^162^

## Appendix 3

### PRISMA Checklist

Download PDF
